# Associations of noise kurtosis, genetic variations in *NOX3* and lifestyle factors with noise-induced hearing loss

**DOI:** 10.1186/s12940-020-0566-3

**Published:** 2020-02-03

**Authors:** Tianyu Zhao, Yinan Wang, Zheng Li, Xiaojun Xu, Song Lei, Liu Huang, Liangwen Xu, Meibian Zhang, Lei Yang

**Affiliations:** 1 Medical School, Hangzhou Normal University, Hangzhou, 310000 China; 2 Medical School, Shihezi University, Shihezi, 832000 China; 3 Ningbo Center for Disease Control and Prevention, Ningbo, 315700 China; 4 Institute of Occupational Health and Radiation Protection, Zhejiang Provincial Center for Disease Control and Prevention, Hangzhou, 310000 China

**Keywords:** Noise, Kurtosis, *NOX3*, Lifestyle, Interaction, Hearing loss

## Abstract

**Background:**

Noise-induced hearing loss (NIHL) is a complex disease caused by environmental and genetic risk factors. This study was to explore the association of noise kurtosis, triphosphopyridine nucleotide oxidase 3 (*NOX3*) and lifestyles with NIHL.

**Methods:**

This case-control study included 307 patients with NIHL and 307 matched control individuals from Zhejiang province of China. General characteristics, noise exposure data, the exfoliated cells of the oral mucosa, and lifestyle details of individuals were collected. The kompetitive allele specific polymerase chain reaction (KASP) method was used to analyze the genotypes of three single nucleotide polymorphisms (SNPs) of *NOX3*.

**Results:**

People who exposed to complex noise had a higher risk of NIHL than those exposed to steady noise (adjusted: OR = 1.806, *P* = 0.002). The GT genotype of additive model and TT + GT genotype of dominant model in *NOX3* rs12195525 decreased the risk of NIHL (adjusted: OR = 0.618, *P* = 0.043; OR = 0.622, *P* = 0.036). Smoking and exposure to high video volume increased the risk of NIHL (adjusted: OR = 1.486, *P* = 0.038; OR = 1.611, *P* = 0.014). Oppositely, regular physical exercise decreased the risk of NIHL (adjusted: OR = 0.598, *P* = 0.004). A positive interaction was found between complex noise and lifestyles including high video volume exposure and no physical exercise in the additive models (RERI = 1.088, *P* < 0.001; RERI = 1.054, *P* = 0.024). A positive interaction was also found between *NOX3* rs12195525 GG genotype and lifestyles including smoking and high video volume exposure in the additive models (RERI = 1.042, *P* = 0.005; RERI = 0.774, *P* = 0.044).

**Conclusions:**

Noise temporal structure, *NOX3* rs12195525 polymorphism, and the three lifestyles of smoking, video volume, and physical exercise were related to the NIHL. There were the interactions between noise temporal structure and the lifestyle of video volume or physical exercise, as well as between *NOX3* and the lifestyle of smoking or video volume. These results provide a theoretical basis for the prevention and genetic testing of NIHL.

## Introduction

Noise-induced hearing loss (NIHL) is irreversible hearing loss caused by cochlear hair cell death combined with synaptic damage resulting from accumulated noise exposure [1, 2]. Usually, NIHL manifests as a temporary or permanent hearing impairment in the 3, 4 and 6 kHz frequency ranges [3]. NIHL is a worldwide occupational health risk and the second most common form of sensorineural hearing loss, after age-related hearing impairment (ARHI) [4]. In the USA and Europe, approximately 30 million employees are exposed to potential noise hazards, and roughly 400–500 million employees are at a risk of developing hearing loss [5]. NIHL is caused by an interaction between genetic and environmental factors, the contributing factors of hearing impairment were categorized into occupational noise exposure, non-occupational noise exposure (such as free time noise exposure and firearm activities), individual susceptibility such as sociodemography (age, gender, ethnicity, and education levels), smoking habit, medical problems (hypertension, diabetes mellitus, hypercholesterolemia, and infections), ototoxic drugs, compliance to hearing protection device (HPD) usage, and knowledge and perception regarding noise and HPD [6–8].

Noise is an important environmental factor causing hearing loss. In the past, noise research was mainly focused on the equivalent continuous sound levels (Leq) and cumulative noise exposure (CNE). These values are formulated using the equal energy hypothesis (EEH), which assumes that the cochlear impact of noise exposure is proportional to the duration of exposure multiplied by the noise intensity. This method is suitable for continuous or steady-state noise, but not for unstable and complex noise. Investigators have found that noise exposures that complex noise produce greater damage to the auditory system than steady noise, in both animals and humans [9, 10]. A complex noise is a non-Gaussian noise consisting of a Gaussian (steady) background noise which is punctuated by a temporally complex series of randomly occurring high-level noise transients. One of the earliest attempts to study non-Gaussian noise using kurtosis to assess the effect on hearing of impulses and impacts was proposed by Erdreich. The advantage of kurtosis would be that all peaks would be accounted for as well as the relative difference between peak and background levels. They proposed that kurtosis statistics could be used to classify the temporal structure of complex noise. The greater the kurtosis, the higher the impulse of the complex noise [11]. This statistic simplifies the time-domain variables affecting hearing (such as the pulse peak value, duration, and the inter-pulse distribution) into one easy-to-calculate parameter which simplifies the classification of complex noise [12]. One purpose of this study is to explore the association between noise kurtosis and hearing loss.

The occurrence of NIHL is based on cochlear hair cell damage and death [13]. *NOX3* is expressed in the inner ear, and is essential for vestibular development and function [14]. In addition to having a physiological role in hearing, *NOX3* could play a role in generating reactive oxygen species (ROS) that react with target molecules within the cochlea, leading to irreversible cochlear cell damage. ROS are implicated in noise, age, and drug-induced cochlear injury. Previous studies have provided evidence that lifestyle habits, such as smoking, drinking, sleeping [15, 16], life and work pressure, physical exercise, and use of ear protection at work are associated with hearing loss [6, 17–19].

This study was designed as a case-control study to analyze the association of noise kurtosis, *NOX3,* lifestyle factors, and their interactions with NIHL in Chinese population occupationally exposed to industrial noise. These results might provide a basis for the genetic screening to prevent noise-induced hearing loss.

## Subjects and methods

### Subjects

A database of manufacturing workers exposed to > 85 dB (A) noise was established. These workers were investigated from 5 factories in Zhejiang Province of East China from October 2017 to December 2018. Inclusion criteria for workers were described as follows:(1) individuals who have worked only in one factory with relatively stable working types; (2) the hearing threshold difference between left and right ears was less than 30 dB at each frequency; (3) no military service; (4) no family history of hearing loss; (5) no ear disease history; and (6) no history of taking ototoxic drugs. The 307 patients with the average hearing threshold > 25 dB at high binaural frequencies (3000, 4000, and 6000 Hz) were selected from the database. The 307 controls with normal hearing, who were matched with gender and age (±3 years), were also selected from the same database.

### Questionnaire survey

Study subjects completed a noise-exposure survey. Collected information included: (1) general characteristics (age, gender, etc.); (2) noise exposure factors (factory, position, work situation, on-the-job time, daily noise exposure duration, etc.); (3) lifestyle factors (smoking, drinking, sleeping time, sleeping duration, video volume, fruits/vegetables intake, physical exercise, life pressure, working pressure, HPDs using). Variables were defined as follows: 1) smoking: average daily cigarette use for 1 year or longer; 2) drinking: average daily alcohol consumption, e.g. white wine ≥50 g, red wine ≥150 g, or beer ≥500 g, and lasting for 1 year or longer; 3) video volume: high video volume ≥ 40% of the maximum volume; low video volume < 40% of the maximum volume; 4) adequate fruits/vegetables intake: average daily intake of fruits and vegetables≥500 g for 1 year or longer; 5) regular physical exercise: average monthly physical exercise for 1–3 times or more for 1 year or longer; (4) overall ear health (history of ear disease, ototoxic drugs intake). The study was approved by the Science Ethics Committee of Hangzhou Normal University (2017LL107). Participants completed study questionnaires and met with trained investigators in a face-to-face interview. Each study participant signed all study consent forms.

### Noise exposure measurement

The International Standardization Organization (ISO) recommends that the A sound level can be used as an indicator of noise health assessment. Eight-hour, continuous equivalent A-weighted sound levels (L_Aeq, 8h_) can be measured with a noise dosimeter. Technical requirements were in accordance with GBZ/T189.8–20 “Workplace Physical Factors Measurement Part 8: Noise”. We used a digital noise dosimeter (ASV5910-R, Hangzhou Aihua Instrument Co., Ltd.) equipped with a 1/4-inch microphone with a 10 Hz to 20 kHz frequency response range and 40–141 dB (A) measurement range. Ambient noise was continuously sampled at 48 kHz. The noise dosimeter was attached to the clothing of the participant at the shoulder by clips, with the microphone pointed up (Additional file 1: Supplementary material 1). The measurement time was 8 h per shift. The noise meter collected noise data every 2 s. After recording noise exposure data, the data was transmitted from the recorder to a computer for subsequent analysis. The noise dosimeter was calibrated using a sound level calibrator (Hangzhou Aihua Instrument, AWA6221B) before and after each sampling cycle.

Cumulative noise exposure (CNE) was used to quantify the noise exposure of each worker according to Formula 1:

1 $$ \mathrm{CNE}=10\lg \left(\sum \limits_{i=1}^n{10}^{\frac{L_{Aeq,\kern0.75em 8h}}{10}}\times {T}_i\right) $$

When the research subjects worked only in same environment, Formula 1 could be simplified to Formula 2:

2 $$ \mathrm{CNE}={L}_{Aeq,8h}+10\mathrm{lgT} $$

In Formulae 1 and 2, CNE unit is dB (A) per year, L_Aeq, 8h_ is the equivalent continuous A-weighted noise exposure level normalized to an 8-h working day, in decibels, occurring over the time interval Ti in years (working years in the i-th work type); with a total of n different noise levels (i.e. different working tasks/environments) that the participants were exposed to during their employment history.

The sampling kurtosis of the continuous 40-s time window of the noise signal during the entire shift was calculated using MATLAB software (Natick, MA). The formula for calculating kurtosis is shown in Formula 3:

3 $$ \upbeta =\frac{\frac{1}{n}\sum \limits_{i=1}^2{\left({x}_i-\overline{x}\right)}^4}{{\left(\frac{1}{n}\sum \limits_{i=1}^2{\left({x}_i-\overline{x}\right)}^2\right)}^2} $$

*x*_*i*_ is the i-th value, x¯ is the sample mean, and β is the noise kurtosis. Kurtosis describes the peakedness of a distribution, which is independent of the overall level and was suggested as a metric of impulsiveness. In theory, the kurtosis of Gaussian noise is 3. However, Gaussian noise is rare in real workplaces. Instead, more noise types are close to Gaussian or complex noise. In this study, 10 of kurtosis was used as the boundary value of Gaussian noise and complex noise, which was supported by other literature [20]. The kurtosis calculation depended on the noise waveform sampling rate and the selected window length. The average kurtosis calculated in a continuous 40-s window was used for the entire shift. Using a 40s window improved computational efficiency while reflecting the dynamic characteristics of complex noise. Additionally, this window established an acceptable kurtosis measurement, based on previous animal data using a similar 48 kHz sampling rate.

### Hearing test and hearing loss diagnosis

In a sound-insulation room with background noise < 25 dB (A), each participant underwent a pure tone test which was performed by an otolaryngologist using a calibrated Madsen OB40 audiometer (GB4854–84). The test was performed on the left and right ears with 500, 1000, 2000, 3000, 4000, 6000, 8000 Hz tones (operating specifications are described in Additional file 1: Supplementary material 2). All subjects were required to be out of the noise environment for at least 16 h before the test. The results of pure tone test were adjusted to gender and age according to the ISO1999:2013 standard. High-frequency noise-induced hearing loss (hNIHL) was diagnosed based on the binaural hearing threshold level at 3, 4, and 6 kHz (HTL _3, 4, 6 kHz_) using Formula 4:

4 $$ {HTL}_{3,4,6 kHz}=\frac{\mathrm{Left}\left({\mathrm{HL}}_{3\mathrm{kHz}}+{\mathrm{HL}}_{4\mathrm{kHz}}+{\mathrm{HL}}_{6\mathrm{kHz}}\right)+\mathrm{Right}\left({\mathrm{HL}}_{3\mathrm{kHz}}+{\mathrm{HL}}_{4\mathrm{kHz}}+{\mathrm{HL}}_{6\mathrm{kHz}}\right)}{6} $$

The binaural high-frequency hearing threshold which was greater than 25 dB was noise-induced hearing loss [21], otherwise is non-binary high-frequency hearing loss.

### SNP selection

The selection of SNPs was mainly through the following methods: (1) literature browsing: search for SNPs reported in previous studies; (2) gene databases browsing: PubMed database (http: //www.ncbi.nlm.nih.gov/snp) and the GeneCard database (http://www.genecards.org). Three SNPs were selected from *NOX3*, including rs3749930, rs12665231, rs12195525 in the exon region. The information of the screened SNPs is shown in Additional file 2: Table S1.

### DNA extraction and genotyping

We used Yongming flocking swabs to collect oral mucosa cells. Swabs were swiped 10 times on the left, right, and lingual mucosa. Then the swab head was broken off into 2 mL centrifuge tubes and stored at − 70 °C. DNA was extracted using Tiangen Oral Mucosa Genomic DNA extraction kits (Tiangen Biotech, Beijing, China) (extraction method listed in the Additional file 1: Supplementary material 3), and genotyped by Hangzhou Hechuan Biological Technology Co., Ltd., using the Kompetitive Allele Specific polymerase chain reaction (KASP) method (Laboratory of the Government Chemist, England). The primer and probe sequences are shown in Additional file 2: Table S2. The following components were combined into the complete reaction mixture: 1) Primer mix consisting of two allele forward primers with different 3′ sequences, reverse primer, and detection primers consisting of two forward primers with unique 5′ sequences; 2) Master mix containing two detection primers with different fluorophores; and 3) DNA template. The assay was performed as follows: PCR reaction 1 – denatured template combines with matching primers in the primer mix and anneals. The detection primer is added to the PCR mix after the extension step. PCR reaction II – Complementary strand synthesis of allele-specific end sequences. PCR reaction III-Signal generation – the levels of allele specific tail increase exponentially and the fluor labelled part of the FRET casette, which is complementary to the tail sequences, bind to them, releasing the fluor and generating a fluorescent signal. In order to control the quality, we randomly selected 10% of the samples to re-classify the genes, and the concordance was 99.5% (rs3749930), 99.8% (rs12665231), 99.7% (rs12195525).

### Statistical analysis

SPSS 24.0 was used for statistical analysis. For general demographic data, normally-distributed continuous variables were expressed as mean ± standard deviation (SD) and were analyzed by Student’s t-tests. Non-normally distributed continuous variables were expressed as median (lower quartile, upper quartile) [M (P_25_, P_75_)] and analyzed by Mann-Whitney U tests. Categorical variables were expressed as frequencies (percentage) and analyzed by χ2 test. Hardy–Weinberg equilibrium was tested using χ2 tests. Association analysis between noise, genotype, lifestyle, and NIHL was performed by binary logistic regression, and reported as odds ratios (ORs) and 95% confidence intervals (95% CI). The interactions between noise, genes, and lifestyle were tested by crossover analysis according to Knol et al. [22]. *P* < 0.05 indicated that the difference was statistically significant and was shown in bold in follow tables. Multiple comparisons were corrected using the Benjamini-Hochberg procedure.

## Results

The subjects included 307 NIHLs and 307 controls. 77.2% of the subjects were male, and 22.8% were female. The median age was 35(30–43) years. The median kurtosis was 17.10(11.17–32.45) in NIHL group, which was significantly higher than that in control group [14.76(9.23–24.95)] (*P* = 0.005). The proportion of people exposed to complex noise was significantly greater than that in the control group (*P* = 0.003). The median threshold shift was 36.83(29.83–49.83) dB in NIHL group, which was significantly higher than that in control group [17.83(14.17–21.00)] dB (*P* < 0.001). All three SNPs in the control subjects were in Hardy–Weinberg equilibrium (*P* > 0.05) (Additional file 2: Table S1). We mainly presented the positive SNP rs12195525 in the Table 1. And the χ2 test found significant difference for the rs12195525 dominant model (*P* = 0.047) (Table 1).

**Table 1:** General characteristics, noise exposure, and genotype distribution between NIHL and control groups

Characteristics	Total (*n* = 614)	NIHL (*n* = 307)	Control (n = 307)	χ^2^/z	*P*
Sex, n (%)				0.000	1.000
Male	474 (77.2)	237 (38.6)	237 (38.6)		
Female	140 (22.8)	70 (11.4)	70 (11.4)		
Age, M (P_25_~P_75_), y	35 (30–43)	36 (30–43)	34 (30–42)	− 1.959	0.050
Education, n (%)					
High School/University	347 (56.5)	168 (27.4)	179 (29.2)	0.802	0.371
Below high school	267 (43.5)	139 (22.6)	128 (20.8)		
Years of exposure, M (P_25_~P_75_), y	3.00 (1.43–6.00)	3.00 (1.20–6.00)	3.00 (2.00–6.00)	−1.002	0.317
Threshold shift, dB	25.00 (17.83–36.96)	36.83 (29.83–49.83)	17.83 (14.17–21.00)	−21.442	<0.001
Kurtosis, M (P_25_~P_75_)	16.10 (9.99–28.33)	17.10 (11.17–32.45)	14.76 (9.23–24.95)	−2.810	0.005
Steady noise	154 (25.1)	61 (9.9)	93 (15.1)	8.875	0.003
Complex noise	460 (74.9)	246 (40.1)	214 (34.9)		
CNE, M (P_25_~P_75_), d B (A)	93.69 (89.48–97.57)	93.73 (89.67–98.24)	93.62 (89.07–96.88)	−1.230	0.219
<85	52 (8.5)	28 (4.6)	24 (3.9)	0.336	0.562
≥ 85	562 (91.5)	279 (45.4)	283 (46.1)		
rs12195525, n (%)				4.041	0.133
Additive					
GG	516 (84.0)	267 (43.5)	249 (40.6)		
GT	87 (14.2)	35 (5.7)	52 (8.5)		
TT	11 (1.8)	5 (0.8)	6 (1.0)		
Dominant					
GG	516 (84.0)	267 (43.5)	249 (40.6)	3.934	0.047
TT + GT	98 (16.0)	40 (6.5)	58 (9.4)		
Recessive					
GG + GT	603 (98.2)	302 (49.2)	301 (49.0)	0.093	0.761
TT	11 (1.8)	5 (0.8)	6 (1.0)		
Allele					
G	1119 (91.1)	568 (46.3)	551 (44.9)	2.910	0.088
T	109 (8.9)	46 (3.7)	63 (5.1)		

We conducted a preliminary exploration of the association between lifestyles and working factors and NIHL. The χ2 test found that the proportion of people smoking, watching video with high volume, and never taking physical exercise in the NIHL group was significantly more than in the control group (*P* < 0.05). However, there were no significant differences between the NIHL and control groups in drinking, sleeping time, sleeping duration, fruits/vegetables intake, life pressure, working pressure and HPDs use (*P* > 0.05) (Table 2).

**Table 2:** Lifestyles and working factors between NIHL and control groups

Lifestyles	Total (*n* = 614)	NIHL (*n* = 307)	Control (*n* = 307)	χ^2^	*P*
Smoking, n (%)					
No	325 (52.9)	150 (24.4)	175 (28.5)	4.086	0.043
Yes	289 (47.1)	157 (25.6)	132 (21.5)		
Drinking, n (%)				1.786	0.181
No	437 (71.2)	211 (34.4)	226 (36.8)		
Yes	177 (28.8)	96 (15.6)	81 (13.2)		
Sleeping time, n (%)				2.975	0.226
~21:00	33 (5.4)	17 (2.8)	16 (2.6)		
21:00~23:00	460 (74.9)	238 (38.8)	222 (36.2)		
23:00~	121 (19.7)	52 (8.5)	69 (11.2)		
Sleeping duration, n (%)				0.062	0.803
~8 h	383 (62.4)	190 (30.9)	193 (31.4)		
8 h~	231 (37.6)	117 (19.1)	114 (18.6)		
Video volume, n (%)				7.453	0.006
Low	149 (24.3)	60 (9.8)	89 (14.5)		
High	465 (75.7)	247 (40.2)	218 (35.5)		
Fruits/vegetables, n (%)				1.085	0.298
Adequate	420 (68.4)	204 (33.2)	216 (35.2)		
Inadequate	194 (31.6)	103 (16.8)	91 (14.8)		
Physical exercise, n (%)				6.745	0.009
Never	418 (68.1)	224 (36.5)	194 (31.6)		
Regular	196 (31.9)	83 (13.5)	113 (18.4)		
Life pressure, n (%)				2.051	0.152
Low	143 (23.3)	64 (10.4)	79 (12.9)		
High	471 (76.7)	243 (39.6)	228 (37.1)		
Working pressure, n (%)				2.440	0.118
Low	113 (18.4)	49 (8.0)	64 (10.4)		
High	501 (81.6)	258 (42.0)	243 (39.6)		
HPDs, n (%)				2.876	0.090
No	297 (48.4)	138 (22.5)	159 (25.9)		
Yes	317 (51.6)	169 (27.5)	148 (24.1)		

The association between noise, genetic models and lifestyles with NIHL was examined, with or without adjustment for gender, age, education and working years, kurtosis, the rs12195525 additive and dominant models, smoking, video volume and physical exercise were found to be significantly associated with NIHL (Table 3). The people who were exposed to complex noise had a higher risk of NIHL compared with those exposed to steady noise (unadjusted: OR = 1.753, 95% CI = 1.209–2.540, *P* = 0.003; adjusted: OR = 1.806, 95% CI = 1.235–2.643, *P* = 0.002). In the rs12195525 additive model, people carrying the GT genotype were found to be less likely to develop NIHL than those with GG genotype (unadjusted: OR = 0.628, 95% CI =0.395–0.996, *P* = 0.048; adjusted: OR = 0.618, 95% CI = 0.388–0.985, *P* = 0.043). Similarly, people carrying the TT + GT genotype of the dominant model were at a lower risk of NIHL than those with the GG genotype (unadjusted: OR = 0.643, 95% CI =0.415–0.997, *P* = 0.048; adjusted: OR = 0.622, 95% CI = 0.400–0.969, *P* = 0.036). Compared with people without smoking, those smoking were more susceptible to NIHL (unadjusted: OR = 1.388, 95% CI =1.010–1.907, *P* = 0.043; adjusted: OR = 1.486, 95% CI = 1.021–2.161, *P* = 0.038). High video volume was 0.681 times more likely to cause NIHL (unadjusted: OR = 1.681, 95% CI =1.156–2.444, *P* = 0.007; adjusted: OR = 1.611, 95% CI = 1.101–2.358, *P* = 0.014). Regular physical exercise posed a lower risk of NIHL compared to no physical exercise (unadjusted: OR = 0.636, 95% CI = 0.452–0.896, *P* = 0.010; adjusted: OR = 0.598, 95% CI = 0.419–0.852, *P* = 0.004).

**Table 3:** Associations of noise exposure, genetic models and lifestyles with risk of NIHL

		Unadjusted OR (95%CI)	Unadjusted *P*	Adjusted OR (95% CI)	Adjusted *P*
Kurtosis	Complex noise/Steady noise	1.753 (1.209–2.540)	0.003	1.806 (1.235–2.643)	0.002
CNE^a^	≥85/<85	0.845 (0.478–1.494)	0.562	0.808 (0.454–1.437)	0.468
rs12195525					
Additive	GT/GG	0.628 (0.395–0.996)	0.048	0.618 (0.388–0.985)	0.043
	TT/GG	0.777 (0.234–2.578)	0.680	0.655 (0.192–2.227)	0.498
Dominant	TT + GT/GG	0.643 (0.415–0.997)	0.048	0.622 (0.400–0.969)	0.036
Recessive	TT/GG + GT	0.831 (0.251–2.751)	0.761	0.700 (0.206–2.378)	0.568
Allele	T/G	0.708 (0.476–1.054)	0.089	0.686 (0.460–1.025)	0.066
Smoking	Yes/No	1.388 (1.010–1.907)	0.043	1.486 (1.021–2.161)	0.038
Drinking	Yes/No	1.269 (0.894–1.802)	0.182	1.273 (0.869–1.865)	0.215
Sleeping time	21:00~23:00/~ 21:00	1.009 (0.498–2.046)	0.980	1.017 (0.495–2.090)	0.963
	23:00~/~ 21:00	0.709 (0.328–1.535)	0.383	0.760 (0.344–1.676)	0.496
Sleeping duration	≥8 h/<8 h	1.043 (0.752–1.445)	0.803	1.150 (0.818–1.615)	0.422
Video volume	High/Low	1.681 (1.156–2.444)	0.007	1.611 (1.101–2.358)	0.014
Fruits/vegetables	Inadequate/Adequate	1.198 (0.852–1.685)	0.298	1.283 (0.906–1.818)	0.161
Physical exercise	Regular/Never	0.636 (0.452–0.896)	0.010	0.598 (0.419–0.852)	0.004
Life pressure	High/Low	1.316 (0.903–1.916)	0.153	1.253 (0.855–1.836)	0.248
Working pressure	High/Low	1.387 (0.919–2.092)	0.119	1.341 (0.883–2.036)	0.169
HPDs	No/Yes	1.316 (0.958–1.807)	0.090	1.330 (0.959–1.846)	0.087

Adjusted for gender, age, education and working years.

^a^Adjusted for gender, age and education.

*P* value <0.05 was considered statistically significant and maintains significance using the Benjamini-Hochberg procedure with the false discovery rate at 0.15

We further analyzed the interactions in noise kurtosis, three lifestyles (smoking, video volume and physical exercise) and rs12195525 (additive and dominant models). The recessive model of SNP is 2 × 2 level when interacting with noise and lifestyle. The dominant model of SNP is 2 × 2 level when interacting with noise and lifestyle. The additive model is 3 × 2 level when interacting with other factors.

Table 4 shows the effect of the interaction between noise kurtosis and lifestyles for the risk of NIHL. After adjustment for gender, age, education and working years, compared with people exposed to steady noise who performed no smoking or regular physical exercise, those exposed to complex noise who performed smoking or no physical exercise were at a higher risk of NIHL (OR = 2.425, 95% CI = 1.393–4.221, *P* = 0.002; OR = 2.656, 95% CI = 1.341–5.261, *P* = 0.005). Within the strata of complex noise, those with smoking, high video volume exposure or no physical exercise had a higher tendency of NIHL than people with no smoking, low video volume exposure or regular physical exercise (OR = 1.585, 95% CI = 1.012–2.484, *P* = 0.044; OR = 1.949, 95% CI = 1.255–3.026, *P* = 0.003; OR = 2.078, 95% CI = 1.368–3.157, *P* = 0.001). Within the strata of smoking, high video volume exposure or no physical exercise, people exposed to complex noise had a higher risk of NIHL than those exposed to steady noise (OR = 2.175, 95% CI = 1.244–3.805, *P* = 0.006; OR = 2.243, 95% CI = 1.441–3.492, *P* < 0.001; OR = 2.242, 95% CI = 1.417–3.546, *P* = 0.001). A positive interaction was found between complex noise and lifestyles including high video volume exposure and no physical exercise in the additive models (RERI = 1.088, 95% CI =0.444–1.732, *P* < 0.001; RERI = 1.054, 95% CI =0.138–1.971, *P* = 0.024).

**Table 4:** Interaction between noise kurtosis and lifestyles for the risk of NIHL

Lifestyles	Steady noise (β < 10)		Complex noise (β ≥ 10)		OR (95% CI) for complex noise within strata of lifestyles	RERI (95%CI)	*P* value
	NIHL/Control (n)	OR (95%CI)	NIHL/Control (n)	OR (95%CI)			
Smoking						0.891(−0.086–1.868)	0.074
No	31/49		119/126				
		1		1.458 (0.863–2.464)	1.458 (0.863–2.464)		
				*P* = 0.159	*P* = 0.159		
Yes	30/44		127/88				
		1.077 (0.554–2.095)		2.425 (1.393–4.221)	2.175 (1.244–3.805)		
		*P* = 0.827		*P* = 0.002	*P* = 0.006		
OR (95% CI) for smoking within strata of kurtosis		1.077 (0.554–2.095)		1.585 (1.012–2.484)			
		*P* = 0.827		*P* = 0.044			
Video volume						1.088 (0.444–1.732)	< 0.001
Low	15/21		45/68				
		1		0.959 (0.446–2.064)	0.959 (0.446–2.064)		
				*P* = 0.916	*P* = 0.916		
High	46/72		201/146				
		0.857 (0.397–1.846)		1.904 (0.944–3.837)	2.243 (1.441–3.492)		
		*P* = 0.693		*P* = 0.072	*P* < 0.001		
OR (95% CI) for high video volume within strata of kurtosis		0.857 (0.397–1.846)		1.949 (1.255–3.026)			
		*P* = 0.693		*P* = 0.003			
Physical exercise						1.054 (0.138–1.971)	0.024
Regular	15/27		68/86				
		1		1.365 (0.665–2.803)	1.365 (0.665–2.803)		
				*P* = 0.397	*P* = 0.397		
Never	46/66		178/128				
		1.238 (0.589–2.599)		2.656 (1.341–5.261)	2.242 (1.417–3.546)		
		*P* = 0.573		*P* = 0.005	*P* = 0.001		
OR (95% CI) for never physical exercise within strata of kurtosis		1.238 (0.589–2.599)		2.078 (1.368–3.157)			
		*P* = 0.573		*P* = 0.001			

Adjusted for gender, age, education and working years. *P* value <0.05 was considered statistically significant and maintains significance using the Benjamini-Hochberg procedure with the false discovery rate at 0.15

Table 5 shows the effect of the interaction between *NOX3* rs12195525 dominant model and lifestyles for the risk of NIHL. After adjustment for gender, age, education and working years, within the strata of GG, people smoking, watching video with high volume or never taking physical exercise were at a higher risk of NIHL (OR = 1.739, 95% CI = 1.152–2.625, *P* = 0.008; OR = 1.740, 95% CI = 1.153–2.626, *P* = 0.008). Within the strata of smoking, or high video volume exposure, people carrying the GG genotype had a higher risk of NIHL than those carrying the TT + GT genotype (OR = 2.507, 95% CI = 1.269–4.955, *P* = 0.008; OR = 1.853, 95% CI = 1.120–3.067, *P* = 0.016). A positive interaction was found between *NOX3* rs12195525 polymorphism and lifestyles including smoking and high video volume exposure in the additive models (RERI = 1.042, 95% CI =0.318–1.765, *P* = 0.005; RERI = 0.774, 95% CI =0.020–1.527, *P* = 0.044).

**Table 5:** Interaction between rs12195525 polymorphism and lifestyles for the risk of NIHL

Lifestyles	Non-risk genotype (TT + GT)		Risk genotype (GG)		OR (95% CI) for risk genotype within strata of lifestyles	RERI (95%CI)	*P* value
	NIHL/Control (n)	OR (95%CI)	NIHL/Control (n)	OR (95%CI)			
Smoking						1.042 (0.318–1.765)	0.005
No	24/30		126/145				
		1		1.076 (0.595–1.947)	1.076 (0.595–1.947)		
				*P* = 0.809	*P* = 0.809		
Yes	16/28		141/104				
		0.705 (0.302–1.646)		1.822 (0.971–3.417)	2.507 (1.269–4.955)		
		*P* = 0.418		*P* = 0.062	*P* = 0.008		
OR (95% CI) for smoking within strata of genotypes		0.705 (0.302–1.646)		1.739 (1.152–2.625)			
		*P* = 0.418		*P* = 0.008			
Video volume						0.774 (0.020–1.527)	0.044
Low	8/13		52/76				
		1		1.087 (0.418–2.821)	1.087 (0.418–2.821)		
				*P* = 0.865	*P* = 0.865		
High	32/45		215/173				
		1.044 (0.383–2.849)		1.905 (0.766–4.734)	1.853 (1.120–3.067)		
		*P* = 0.933		*P* = 0.165	*P* = 0.016		
OR (95% CI) for high video volume within strata of genotypes		1.044 (0.383–2.849)		1.740 (1.153–2.626)			
		*P* = 0.933		*P* = 0.008			
Physical exercise						0.124(−1.289–1.537)	0.864
Regular	13/24		70/89				
		1		1.759 (0.819–3.778)	1.759 (0.819–3.778)		
				*P* = 0.147	*P* = 0.147		
Never	27/34		197/160				
		1.924 (0.802–4.617)		2.807 (1.349–5.843)	1.449 (0.834–2.517)		
		*P* = 0.143		*P* = 0.006	*P* = 0.188		
OR (95% CI) for never physical exercise within strata of genotypes		1.924 (0.802–4.617)		1.605 (1.091–2.361)			
		*P* = 0.143		*P* = 0.016			

Adjusted for gender, age, education and working years. *P* value <0.05 was considered statistically significant and maintains significance using the Benjamini-Hochberg procedure with the false discovery rate at 0.15

Table 6 shows the effect of the interaction between noise kurtosis and *NOX3* rs12195525 dominant model for the risk of NIHL. After adjustment for gender, age, education and working years, compared with people exposed to steady noise who carried the TT + GT genotype, those exposed to complex noise who carried the GG genotype were at a higher risk of NIHL (OR = 4.884, 95% CI = 1.603–14.879, *P* = 0.005). Within the strata of TT + GT genotype, people who were exposed to complex noise had a higher tendency of NIHL than those exposed to steady noise (OR = 3.429, 95% CI = 1.051–11.187, *P* = 0.041). Among the people carrying the GG genotype, those exposed to complex noise demonstrated a higher risk of NIHL than those exposed to steady noise (OR = 1.726, 95% CI = 1.146–2.600, *P* = 0.009). The interaction between noise kurtosis and rs12195525 polymorphism was not found in the additive models (*P* value of RERI > 0.05).

**Table 6:** Interaction between noise kurtosis and rs12195525 polymorphism for the risk of NIHL

Genotypes	Steady noise (β < 10)		Complex noise (β ≥ 10)		OR (95% CI) for complex noise within strata of genotype	RERI (95%CI)	*P* value
	NIHL/Control (n)	OR (95%CI)	NIHL/Control (n)	OR (95%CI)			
rs12195525						−0.307(−2.884–2.270)	0.815
Non-risk genotype (TT + GT)	4/16		36/42				
		1		3.429 (1.051–11.187)	3.429 (1.051–11.187)		
				*P* = 0.041	*P* = 0.041		
Risk genotype (GG)	57/77		210/172				
		2.961 (0.940–9.332)		4.884 (1.603–14.879)	1.726 (1.146–2.600)		
		*P* = 0.064		*P* = 0.005	*P* = 0.009		
OR (95% CI) for risk genotype within strata of kurtosis		2.961 (0.940–9.332)		1.495 (0.911–2.455)			
		*P* = 0.064		*P* = 0.112			

Adjusted for gender, age, education and working years.

*P* value <0.05 was considered statistically significant and maintains significance using the Benjamini-Hochberg procedure with the false discovery rate at 0.15.

Additional file 2: Table S3 shows the effect of the interaction between *NOX3* rs12195525 dominant model and lifestyles for the risk of NIHL in the 2 × 3 level analysis. After adjustment for gender, age, education and working years, among the people carrying the GG genotype, those who performed smoking or no physical exercise demonstrated a higher risk of NIHL than those who performed no smoking or regular physical exercise (OR = 1.739, 95% CI = 1.152–2.625, *P* = 0.008; OR = 1.605, 95% CI = 1.091–2.361, *P* = 0.016). The interaction between rs12195525 polymorphism and lifestyles was not found in the additive models (*P* value of RERI > 0.05). Because of insufficient samples, the interactions of rs12195525 dominant model and noise kurtosis, video volume in the 2 × 3 level analysis were not examined.

## Discussion

NIHL is a complex disease which is caused by the interaction of gene and environment. Nonetheless, NIHL mainly depends on the degree of biological damage caused by noise exposure. Our focus this time was on noise characteristics—kurtosis. It can describe the characteristics of noise pulse in a simple and effective way, and then distinguish steady and complex noise. Various time-domain variables, including pulse interval distribution, duration, noise pulse peak can be transformed to simple variables by kurtosis, which simplifies noise classification greatly. Animal experiments and some population epidemiological investigations demonstrate that kurtosis can be used as an auxiliary energy parameter to effectively assess the biological effects of complex noise [9, 10]. Further, kurtosis can be used to compensate for hearing loss caused by complex noise in existing international standards. In this study, the median noise kurtosis of the NIHL group was greater than that of the control group, as well as the proportion of workers exposed to complex noise. Multivariate analysis adjusted to age, gender, education, and working years, demonstrated the NIHL risk in people exposed to complex noise was 0.806 times higher than people exposed to steady noise. These results correspond with previous studies of Zhao, YM, Xie, HW, et al. [10, 12]. Noise kurtosis injures the auditory system by disruption of metabolism and direct mechanical force [10]. However, we have not found the association between CNE and NIHL in our study.

*NOX3* encodes NADPH oxidase, which produces superoxide and other reactive oxygen species (ROS) during oxidative stress or signal transduction. Increased NADPH activity can initiate apoptosis or necrosis of inner ear hair cells [15, 23]. The rs12195525 locus is located in the coding region of *NOX3*, and there are two mutations, G → A and G → T, which cause arginine (Arg) to become a terminator (Ter) and arginine to arginine (synonymous mutation). At present, there is no study on the association between rs12195525 and NIHL, in East Asian or European populations. The G and T alleles in this study population were 91.1% and 8.9%, respectively, which were not consistent with European population G (89.6%) and T (10.4%) alleles, but were basically consistent with East Asian population G (88.3%) and T (11.7%) alleles (https://www.ncbi.nlm.nih.gov/snp/rs12195525#frequency_tab), which indicating that there were possibly racial and ethnic differences in the rs12195525 locus allele frequency. The results of this study showed that there was rs12195525 polymorphism in Chinese, and there were three genotypes of GG, GT and TT in NIHL group and control group. The distribution of GG, GT + TT genotype frequencies in the dominant model was statistically significant in the two groups. In the additive model, the GG genotype was used as a reference for univariate logistic regression analysis. The results showed that the risk of NIHL in people with CT genotype was 0.628 times than that of GG genotype. Perform further multivariate logistic regression analysis, after adjustment of the age, gender, education, and working years, the risk above became 0.618 times higher. The GG genotype was also used as a reference in the dominant model while performing univariate logistic regression analysis, which showed that the risk of NIHL in people carrying the TT + GT genotype was 0.643 times higher than that of those carrying the GG genotype. And perform further multivariate logistic regression analysis, after adjustment of the above confounding factors, the risk became 0.622 times higher. It illustrated that the GT genotype of dominant model and the TT + GT genotype of additive model are the protective factors of NIHL. The NOX system is the main ROS producer in cells. Overactive NOS system proteins produce elevated ROS levels, which damages cells [15]. The *NOX3*-encoded NADPH oxidase is highly expressed in the inner ear, especially in the cochlear and vestibular sensory epithelium [24]. Previous studies demonstrated that *NOX3* mutations do not affect normal hearing in mice [14], but Lavinsky et al. reported that *NOX3* mutants are more sensitive to NIHL [25]. Although the rs12195525 G → T polymorphism produces a synonymous mutation where the encoded amino acid is still arginine, it may affect NADPH oxidase expression such as transcription, translation, folding, secretion and post-translational modification [26–28]. Historically, mutations that do not cause amino acid sequence changes were not believed to cause disease. However, increasing evidence suggests that synonymous mutations can affect the amino acid translation efficiency [29]. Thus, the rs12195525 SNP could affect noise-induced hearing loss by altering NADPH oxidase expression.

This study analyzed the association of lifestyles including smoking, drinking, sleeping time, sleeping duration, video volume, fruits and vegetables intake, physical exercise, life and working pressure and HPDs using with NIHL. With or without adjusting gender, age, education and working years, smoking, video volume exposure and physical exercise were found to be significant in the occurrence of NIHL. We found that smoking, high video volume, and no physical exercise increased the risk of NIHL, consistent with most previous studies [30–32]. Smoking affects blood flow to the cochlea and increases blood carboxyhemoglobin levels. Therefore, smoking may reduce oxygen transport to auditory cells [33, 34], causing anoxia and increasing the risk of hearing loss. A study by Su et al. suggests that high-volume noise exposure may lead to hearing loss via a mechanism involving reduced cochlear oxygen tension during and after noise exposure [35]. In addition, many studies have shown that physical exercise reduces the risk hearing loss. Too little exercise affects blood, oxygen, and nutrient flow to the cochlea, which degrades the stria vascularis (SV). Blood vessels in the SV are essential for transporting nutrients such as oxygen and glucose to the cochlea [36].

In our case-control study, we used the relative excess risk of interaction (RERI) of the additive interaction model as a standard measure. The RERI value as well as its 95% confidence interval and *P* value were reported here. When RERI = 0, there is no interaction between the two factors; when RERI>0, there is a positive interaction between the two factors; when RERI<0, there is a negative interaction between the two factors. Here we selected positive results of the multivariate analysis, which included noise kurtosis, three lifestyles (smoking, video volume and physical exercise), and *NOX3* rs12195525 (additive and dominant models) for the interaction analysis.

For the first time, we determined that noise kurtosis, genetics, and lifestyle interact to affect NIHL. In the additive interaction model. Our results showed that there were positive interactions between noise kurtosis with video volume and physical exercise. This results suggested that the risk of NIHL in people exposed to complex noise (β > 10) who watched video with high volume or had no physical exercise is greater than the combined risk in people exposed to complex noise alone and those watching video with high volume or having no physical exercise alone. In addition, we also found the positive interactions between *NOX3* with smoking and video volume. The risk of NIHL in people carrying the GG genotype of rs12195525 who performed smoking or high video volume exposure is higher than the combined risk in people carrying the rs12195525 GG genotype alone and those with smoking or high video volume exposure alone. In the interaction tables recommended by Knol, we also found that among the people exposed to complex noise, those with smoking, high video volume exposure or no physical exercise had a higher risk of NIHL. Similarly, among people performing smoking, high video volume exposure or no physical exercise, those exposed to complex noise (β ≥ 10) were at higher risk of NIHL than exposed to steady noise (β < 10). What’s more, among the people carrying the *NOX3* rs12195525 GG genotype, those with smoking and high video exposure had a higher risk of NIHL. Among the people with smoking and high video exposure, those carrying the *NOX3* rs12195525 GG genotype were at a higher risk of NIHL. We didn’t find the interactions between noise kurtosis and *NOX3*. However, it was found that among the people carrying the rs12195525 GG genotype, those exposed to complex noise had a higher risk of NIHL. We didn’t find the interactions of 2 × 3 level analysis between *NOX3* rs12195525 with other factors. However, it was observed that among the people carrying the GG genotype, those with smoking and no physical exercise had a higher risk of NIHL.

NIHL is a complex disease which is caused by the interaction of gene and environment. We demonstrate that rs12195525 (*NOX3*), lifestyle, and noise exposure are each associated with NIHL. Additionally, our results revealed complex interactions between genes, environment, and lifestyle. We can speculate that the unfavorable environment and genetic susceptibility will increase the risk of NIHL. In the future, it is necessary to increase the sample size and combine laboratory studies to clarify the specific mechanisms of interaction between mutations and environmental factors.

Our study had some advantages. First, we explored the interactions of noise kurtosis, gene expression, and lifestyle factors for the first time. Kurtosis describes the noise pulse and distinguishes steady and complex noise. Second, we used the interaction presentation tables recommended by Knol et al. to report the RERI interaction index, OR value, and 95% CI. This analysis combined the number of subjects in the NIHL and control groups with the stratified analysis results. Thus, the table-based analysis provided a more complete understanding of the raw data characteristics. However, there were some limitations to our study. First, the correlation between window time length and hearing loss needed further study because the types and data of noise sources were not sufficiently abundant in this study. Second, we were unable to obtain data regarding other confounding factors, such as hypertension and diabetes because of technical reasons, which might influence the results. Third, crossover analysis required factors to be two-category variables. Thus, the crossover analysis was only useful for analyzing interactions between single genetic and environmental factors. Fourth, self-reported data might be inaccurate, thereby affecting the study effectiveness. Finally, the sample size was insufficient. Further functional studies are warranted to validate our results with larger samples and to investigate the molecular mechanisms underlying the interactions identified in our study.

## Conclusion

In conclusion, noise kurtosis (noise temporal structure), *NOX3* rs12195525 polymorphism and three lifestyles (smoking, video volume and physical exercise) were found to be associated with NIHL. Our results also found the interactions between noise kurtosis and video volume or physical exercise, as well as *NOX3* and smoking or video volume. These results provide a theoretical basis for the prevention and genetic testing of NIHL.

## Supplementary information

**Additional file 1: Supplementary material 1.** Code for the Wearing of Noise Measuring Instruments. **Supplementary material 2.** Technical Specifications for Audiometry. **Supplementary material 3.** DNA Extraction of Mucosal Exfoliated Cells.

**Additional file 2: Table S1.** Gene information. **Table S2.** Primer information A. Table S2 Primer information B. **Table S3**. Interaction between rs12195525 polymorphism and lifestyles for the risk of NIHL.

## Data Availability

Please contact author for data requests.

## Abbreviations

ARHI: Age-related hearing impairment
CI: Confidence interval
CNE: Cumulative noise exposure
ISO: International Standardization Organization
KASP: Kompetitive Allele Specific polymerase chain reaction
NIHL: Noise-induced hearing loss
*NOX3*: Triphosphopyridine Nucleotide Oxidase 3
OR: Odds ratio
RERI: Relative excess risk of interaction
ROS: Reactive oxygen species
SD: Standard deviation
SNP: Single nucleotide polymorphism
SV: Stria vascularis
