# Supplementary material for: Associations of noise kurtosis, genetic variations in NOX3 and lifestyle factors with noise-induced hearing loss
Source: Environ Health. 2020 Feb 3;19:13. doi: 10.1186/s12940-020-0566-3 (PMC6998835; doi:10.1186/s12940-020-0566-3)
Supplement: Supplementary file 1 — Additional file 1: Supplementary material 1. Code for the Wearing of Noise Measuring Instruments. Supplementary material 2. Technical Specifications for Audiometry. Supplementary material 3. DNA Extraction of Mucosal Exfoliated Cells. [file 12940_2020_566_MOESM1_ESM.docx]

**Supplementary material 1**

**Code for the Wearing of Noise Measuring Instruments**

Dear workers,

Hello!

In order to find out the noise value of workers' working environment, protect workers' health and promote the environment of workers' working place, we have tracked the noise monitoring of your factory's noise workers with the strong support of the company leaders. For the monitoring quality, we hope you can be sure to do the following:

1. The employees who wear the instrument are not allowed to take off, and must wear it until the monitor or our staff to take, no matter working, having a meal, going to the toilet or doing other things.

2. The microphone of the instrument is valuable and fragile. Workers should be careful and protect it from impact.

3. The workers or others are not permitted to bump, squeeze, touch or look at the instrument. Moving the button, switch or open the instrument package are also not allowed.

Maybe your hard work will be good for the improvement of your working conditions in the future. We hope you can cooperate with us.

Thank you very much!

**Supplementary material 2**

**Technical Specifications for Audiometry**

1. Hearing measurement objective: to reflect workers' listening threshold level.

2. Requirements for audiometry equipment: electric audiometry equipment that meets the national occupational health standards and is within the validity period of quality inspection; There is a special electric audiometry and soundproof room, and the noise level of the soundproof indoor environment shall meet the minimum threshold of at least 0dB.

3. Hearing measurement process

3.1 Registration and application of workers.

3.2 Wait quietly in line outside the hearing measurement room.

3.3 Before the new survey starts, guide workers into the survey room and explain the matters needing attention. Explanation contents include: A. instruct workers to sign the informed consent of the project: B. wear earphones correctly and do not take them off during the measurement: C. Start self-study listening and press the button immediately when you hear the "dripping" sound; D. Do not press the button until you hear the "dripping" sound; E. If there is any discomfort during the measurement, inform the measuring personnel immediately.

4. Audiometry:

A. The test tone shall be given for 1-2 seconds per time with irregular intervals, but each interval shall not be shorter than the duration of the sound given.

B. Step 1: elevating method: make the subjects adapt to 1000Hz 40dBHL; if there is no reaction, increase the test sound by 10dB until they can make a response; drop the test sound down by 10dB, and then gradually increase the test sound until the response made. Rising method: after adapting to the test sound, drop 10dB before measuring, and if the subjects hear nothing, increase the sound level by 5dB first gear until a response.

C. Second step: lifting method: after reacting, add 5dB, then decrease 5dB step by step until there is no reaction, decrease 5dB, then increase 5dB step by step, repeating that three times, average the minimum date of three times when the subjects respond to the sound, which is the hearing threshold level; Rising method: A response, drop 10dB, and then increase 5dB step by step, repeating that 5 times, in the same hearing level response is the hearing threshold level.

D. Step 3: testing the next frequency, start at 10dB below the threshold just measured, and then measure the frequency in turn.

E. Step 4: measure the other ear by the same method.

**Supplementary material 3**

**DNA Extraction of Mucosal Exfoliated Cells**

**1. The collection and processing of oral mucosal exfoliated cells**

Use the Yongming flocking swabs to collect the exfoliated cells of the oral mucosa, and wipe back and forth 10 times on the left, right and lingual mucosa of the subject’s oral cavity with a slight gravity. The head of the swabs should be broken and put into the 2ml EP tubes. Then we store them in a refrigerator at -70℃ for later use.

**2. Genomic DNA extraction and genotyping**

Extracting sample genomic DNA by DNA extraction method of column exfoliated oral mucosa cells.

The experimental steps are as follows:

(1) Sampling: Wipe 6-10 times on the inner wall of the mouth with a buccal swab and dry it for 2 hours. To ensure that the sample is not contaminated by food or drink, please do not eat or drink within 30 minutes before sampling.

(2) The swab that has been wiped on the inner wall of the oral cavity should be cut out from the rod with scissors, then be put into a 2ml centrifuge tube. And we add 400μl Buffer PBS to the centrifuge tube. If RNA-free genomic DNA is required, 20μl 10 mg/ml RNase A solution can be added to the centrifuge tube.

(3) Add 400μl Buffer ACL, 200μl Buffer CL and 20μl Proteinase K into the centrifuge tube, shake and mix it. Then bath it in water at 65℃ for 10 min, mix occasionally.

(4) Add 400μl absolute ethanol into the centrifuge tube, and mix it well. Then transfer all the solution and precipitate to the silica gel membrane adsorption column (adsorption column should be put into the collection tube), centrifuge at 10,000rpm for 1 min, and drain the solution in the collection tube. After that, place the column back into the collection tube. The maximum capacity of the adsorption column is 750μl, the adsorption column should be used again; if the concentration of the genomic DNA is not required very high in the subsequent experiments, the adsorption column can be used only once.

(5) Add 500μl Wash Solution into the adsorption column, centrifuge at 10,000rpm for 1 min, drain the solution in the collection tube, and place the adsorption column back into the collection tube. Before using the Wash Solution, please check if the solution is added with absolute ethanol.

(6) Repeat step 5 again.

(7) Place the adsorption column back into the collection tube, and centrifuge at 12000rpm for 2 min. This step must not be omitted, otherwise the residual ethanol will seriously affect the subsequent experiments.

(8) Take out the adsorption column, place it into a new 1.5 ml centrifuge tube, and add 50μl TE Buffer to the center of the adsorption membrane. After standing for 3 min, centrifuge at 12000 rpm for 2 min. Then the DNA solution can be stored at -20°C or used directly in the subsequent experiments. If you want to improve the productivity of the DNA, you can repeat step 8 or preheat the TE Buffer at 60°C.

(9) Use a NanoDrop spectrophotometer to test the concentration and purity of the product. Please ensure the ratio of OD_260_/OD_280_ is between 1.7 and 2.0, meanwhile the ratio of OD_260_/OD_230_ is greater than 2.0 and the concentration is 20-50ng/μl.

(10) Repeat the extraction of unqualified samples until all samples are qualified. Then sent them to the company for genotyping.
