# Supplementary material for: Associations of noise kurtosis, genetic variations in NOX3 and lifestyle factors with noise-induced hearing loss
Source: Environ Health. 2020 Feb 3;19:13. doi: 10.1186/s12940-020-0566-3 (PMC6998835; doi:10.1186/s12940-020-0566-3)
Supplement: Supplementary file 2 — Additional file 2: Table S1. Gene information. Table S2. Primer information A. Table S2 Primer information B. Table S3. Interaction between rs12195525 polymorphism and lifestyles for the risk of NIHL. [file 12940_2020_566_MOESM2_ESM.docx]

**Table S1** Gene information

| SNP | Gene | Chromosome | Position | Functional consequence | Allele | MAF^a^ | MAF^b^ | HWE p |
| --- | --- | --- | --- | --- | --- | --- | --- | --- |
| rs3749930 | NOX3 | 6 | 155440112 | Missense Variant | G>T | 0.197 | 0.477 | 0.662 |
| rs12665231 | NOX3 | 6 | 155395463 | Missense Variant | T>C | 0.188 | 0.243 | 0.126 |
| rs12195525 | NOX3 | 6 | 155454846 | Missense Variant | G>A,T | 0.129 | 0.104 | 0.103 |
| MAF：Minor allele frequency; HWE: Hardy–Weinberg equilibrium;  a: 1000genomes; b: Data form this study; | | | | | | | | |

**Table S2** Primer information A

| ID | Primer Allele FAM | Primer Allele HEX | Primer Common |
| --- | --- | --- | --- |
| rs3749930 | ACACAACCACTGAATTGCTAAGGAC | AACACAACCACTGAATTGCTAAGGAA | CAGACCGGTGACGCCTGCTATT |
| rs12665231 | AGCTAAGGAAGTATGGTATAGTGTACT | GCTAAGGAAGTATGGTATAGTGTACC | CCCAGTTGGGAACTGGTGAATAATAATTA |
| rs12195525 | GTTCCTCTTATGAATGAAATAAGGTTTCA | GTTCCTCTTATGAATGAAATAAGGTTTCG | CTGTGCCTGAATTTTAACTGCATGCTAAT |

**Table S2** Primer information B

| ID | Allele FAM | Allele HEX | Sequence | CG%_FAM | CG%_HEX | CG% Common |
| --- | --- | --- | --- | --- | --- | --- |
| rs3749930 | G | T | GACCGGTGACGCCTGCTATT[G/T]TCCTTAGCAATTCAGTGGTT | 44 | 38.5 | 59.1 |
| rs12665231 | T | C | GGAAGTATGGTATAGTGTAC[T/C]GTTCACAATAGTTAATTATT | 37 | 42.3 | 37.9 |
| rs12195525 | A | G | TATGAATGAAATAAGGTTTC[A/G]ACTGACAGGTATTAGAATTA | 30 | 32.3 | 37.9 |

| **Table S3** Interaction between rs12195525 polymorphism and lifestyles for the risk of NIHL | | | | | | | | | |
| --- | --- | --- | --- | --- | --- | --- | --- | --- | --- |
| Lifestyles | | Genotype | | | | | | ORs (95%CI) for GT within strata of lifestyles | ORs (95%CI) for GG within strata of lifestyles |
|  |  | TT | | GT | | GG | |  |  |
|  |  | NIHL/control(n) | OR (95%CI) | NIHL/control(n) | OR (95%CI) | NIHL/control(n) | OR (95%CI) |  |  |
| Smoking | No | 4/4 |  | 20/26 |  | 126/145 |  |  |  |
|  |  |  | 1 |  | 1.138(0.243-5.335);  *P* =0.870 |  | 1.201(0.284-5.074);  *P* = 0.804 | 1.138(0.243-5.335);  *P* =0.870 | 1.201(0.284-5.074);  *P* = 0.804 |
|  | Yes | 1/2 |  | 15/26 |  | 141/104 |  |  |  |
|  |  |  | 0.742 (0.044-12.357); *P*= 0.835 |  | 0.790(0.162-3.848);  *P* = 0.771 |  | 2.036 (0.465-8.923);  *P* = 0.345 | 1.339 (0.101-17.708);  *P* = 0.825 | 2.758(0.246-30.982);  *P* = 0.411 |
| ORs (95%CI) for smoking within strata of genotype | |  | 0.742 (0.044-12.357);  *P* = 0.835 |  | 0.594(0.214-1.645);  *P* = 0.316 |  | 1.739(1.152-2.625);  *P* = 0.008 |  |  |
| RERI (95%CI) | | | | -0.0887(-2.6072-2.4298); *P* = 0.945 | | 1.094(-0.809-2.996);  *P* = 0.260 | |  |  |
| Physical exercise | Regular | 1/1 |  | 12/23 |  | 70/89 |  |  |  |
|  |  |  | 1 |  | 0.727(0.041-13.012);  *P* = 0.828 |  | 1.303(0.077-22.041);  *P* = 0.854 | 0.727(0.041-13.012);  *P* = 0.828 | 1.303(0.077-22.041);  *P* = 0.854 |
|  | Never | 4/5 |  | 23/29 |  | 197/160 |  |  |  |
|  |  |  | 1.247 (0.056-27.831);  *P* = 0.889 |  | 1.457(0.082-25.757);  *P* = 0.797 |  | 2.078(0.124-34.855);  *P* = 0.611 | 0.924(0.184-4.642);  *P* = 0.924 | 1.626(0.418-6.329);  *P* = 0.483 |
| ORs (95%CI) for never physical exercise within strata of genotype | |  | 1.247 (0.056-27.831);  *P* = 0.889 |  | 1.976(0.684-5.710);  *P* = 0.208 |  | 1.605(1.091-2.361);  *P* = 0.016 |  |  |
| RERI (95%CI) | | | | 0.482(-3.657-4.621); *P* = 0.819 | | 0.526 (-6.457-7.510); *P* = 0.883 | |  |  |
| Adjusted for gender, age, education and working years. | | | | | | | | | |
| *P* value < 0.05 was considered statistically signiﬁcant and maintains signiﬁcance using the Benjamini-Hochberg procedure with the false discovery rate at 0.15. | | | | | | | | | |
